# Copper Oxide Nanomaterials Prepared by Solution Methods, Some Properties, and Potential Applications: A Brief Review

**DOI:** 10.1155/2014/856592

**Published:** 2014-12-16

**Authors:** Thi Ha Tran, Viet Tuyen Nguyen

**Affiliations:** ^1^Hanoi University of Mining and Geology, Co Nhue, Tu Liem, Hanoi 130503, Vietnam; ^2^College of Science, Vietnam National University, 334 Nguyen Trai, Thanh Xuan, Hanoi 120034, Vietnam

## Abstract

Cupric oxide (CuO), having a narrow bandgap of 1.2 eV and a variety of chemophysical properties, is recently attractive in many fields such as energy conversion, optoelectronic devices, and catalyst. Compared with bulk material, the advanced properties of CuO nanostructures have been demonstrated; however, the fact that these materials cannot yet be produced in large scale is an obstacle to realize the potential applications of this material. In this respect, chemical methods seem to be efficient synthesis processes which yield not only large quantities but also high quality and advanced material properties. In this paper, the effect of some general factors on the morphology and properties of CuO nanomaterials prepared by solution methods will be overviewed. In terms of advanced nanostructure synthesis, microwave method in which copper hydroxide nanostructures are produced in the precursor solution and sequentially transformed by microwave into CuO may be considered as a promising method to explore in the near future. This method produces not only large quantities of nanoproducts in a short reaction time of several minutes, but also high quality materials with advanced properties. A brief review on some unique properties and applications of CuO nanostructures will be also presented.

## 1. Introduction

In recent years, nanostructures of transition metal oxides have gained a great attention from material scientists and engineers due to their different properties compared with the corresponding bulk counterparts, which in turn provides promising applications in various fields of technology. Preparation of high quality nanostructures of defined, controllable size and morphology is a critical requirement in order to develop nanodevices or other different applications for catalyst, sensing, pharmacy [1–7], and so on.

CuO, categorized into transition metal oxide group, is a p type, narrow bandgap semiconductor. It has monoclinic structure and many interesting characteristics: super thermal conductivity, photovoltaic properties, high stability, and antimicrobial activity. Due to such exclusive properties, CuO can be used in many technological fields, for example, active catalyst [1], gas sensor [4, 6], high efficiency thermal conducting material [7], magnetic recording media [8], with very good selectivity, or solar cell applications [9].

In addition to some shared properties of metal oxide nanostructures, such as TiO_2_, ZnO, WO_3_, and SnO_2_, CuO nanostructures have other unique magnetic and super-hydrophobic [10] properties. Furthermore, these nanostructures show very promising applications in heterogeneous catalysis in the complete conversion of hydrocarbons into carbon dioxide, enhancement of thermal conductivity of nanofluid, nanoenergetic materials, and super-hydrophobic surfaces or anode materials for lithium ion batteries (LIBs). However, this material has not got attention of scientists at right level until recent years. Compared with other oxides of transition metal such as Fe_2_O_3_, TiO_2_, and ZnO only few reports have described the synthesis strategies adopted for CuO nanostructures along with the introduction of their related applications.

In this paper we would like to systematically discuss the effect of some factors in solution based process on the nanoproducts rather than focus on each individual synthesis process. For each factor, critical comments will be provided based on our knowledge and related research experience. Some properties and potential applications of CuO nanostructures reported in the literature to date will also be presented. This review aims to provide a critical discussion of the synthesis of CuO nanostructures. The potentials of CuO nanostructures as functional components for fabrication of micro/nanodevices are also evaluated and highlighted. In particular, we focus on the fundamental properties and various nanostructured forms of CuO that have been reported in the literature to date and summarize the various synthetic strategies. Precisely controlling the synthetic strategies is very critical because it helps to obtain CuO nanostructures with manageable dimensions and morphology which is crucial for obtaining corresponding unique properties. This in turn enables a variety of promising applications that would not be possible for bulk material. The aim of this review is to provide critical discussion about important factors that can affect morphologies and size of CuO nanoproducts prepared by solution methods. The review hopefully can contribute some useful information that helps to better understand the relation between synthetic process, final morphology, and the properties of corresponding CuO nanostructures. A better understanding of the fundamental properties of CuO nanostructures is essential for their application as building blocks for functional devices.

## 2. Synthesis of CuO Nanostructures

The development of synthetic methods has been widely accepted to contribute an important part to fundamental study for understanding properties and realizing applications of nanoscale materials. It allows material scientists to control different parameters of the products such as shape, particle size, size distribution, and composition. Numerous methods such as thermal evaporation, sonochemical, sol-gel, hydrothermal, and electrochemical methods [2, 11–15] and microwave irradiation approach [16–18] have been developed to synthesize CuO nanostructures with diverse morphologies, sizes, and dimensions using various chemical, physical, or chemistry physics combined strategies. This paper focuses only on direct solution methods to successfully prepare copper (II)-oxide (Cu=O) nanostructures with different sizes, shapes, some of their properties and different applications in daily life and technology. The main reason why we would like to limit our considerations only to the wet chemical methods is that these approaches offer many advantages compared with physics synthesis processes such as the possibility to use low cost and high-throughput equipment, low wastage of raw materials, high uniformity of size and shape of the nanoproduct, and, finally, potential deployment of large scale production with low capital investment.

In this review, we would like not to present the synthetic strategies in detail but rather to discuss the effect of major factors of the synthetic process such as solvents, starting materials, and additive materials on CuO nanoproducts.

A typical direct solution method to prepare CuO nanostructures usually involves the following steps: preparing the precursor solution, modification of nanoproducts with additives or surfactants, heat treatment, and washing and drying process.

CuO nanoproducts are prepared according to the following equations:

(1) Copper  salt+Alkaline  hydroxide  ⟶CuOH2+Salt  of  alkaline  metal

(2) $$\begin{array}{c} {\text{Cu}\left(\text{OH}\right)}_{2}⟶\text{CuO}+{\text{H}}_{2}\text{O} \end{array}$$

**Figure 1:**
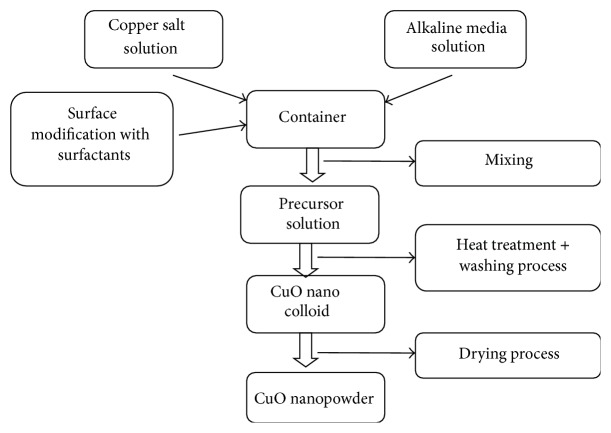
Schematic diagram of a typical direct solution synthesis of CuO nanostructures.

The entire process was summarized by the diagram in Figure 1.

In the next part, we will discuss in detail possible effects of each factor on the morphology and properties of final products.

### 2.1. Effect of Starting Materials

#### 2.1.1. Solvent

Solvent is one of the most important components of wet chemical methods as solvent has a crucial effect on the product. Due to the critical role of solvent, it is sometimes used to name a particular wet chemical approach, for example, alcohol-thermal synthesis or DMSO (dimethyl sulfoxide) route. Two primary criteria for the solvents used to synthesize CuO nanostructures are as follows: (i) they dissolve copper and alkali hydroxide compounds and (ii) they can be washed away easily or decomposed during the washing and drying process without leaving any detrimental impurities or residues in the final nanoproduct. There are many secondary factors that great attention should be paid for the synthesis process such as viscosity, surface tension, volatility, reactivity, toxicity, and cost.

In order to dissolve well copper salts and alkali hydroxide, which are ionic compounds, polar solvents are normally used for synthesizing CuO nanostructures. Water is certainly the cheapest, safest, and most environmentally friendly solvent. In fact, in most of the reports, CuO nanostructures were prepared by utilizing water as solvent; however, there are only few reports of using water solely without any additives or surfactants (see Table 1). Preparation of nanostructures in water without any additives normally leads to large (several hundreds of nanometers) and nonuniform size and shape particles or complex structures like 3D flower-like structures. These results imply that water itself has some disadvantages that make scientists prefer to use other organic solvents such as alcohols of different carbon chain length or number of functional groups (OH). The main disadvantage of using water as solvent is that water tends to promote the coarsening process during the growth of nanostructures prepared from liquid phase. Coarsening involves the growth of larger crystals at the expense of smaller crystals and is governed by capillary effects. Since the chemical potential of a particles increases with decreasing particle size, the equilibrium solute concentration for small particles is much higher than that for large particles or, in other words, larger particles are more energetically favored than smaller particles. The resulting concentration gradients lead to transport of solute from the small particles to the larger particles. The rate law for this process, derived by Lifshitz, Slyozov, and Wagner (LSW) [19], is given by

(3) $$\begin{array}{c} {\overset{-}{r}}^{3}-{\overset{-}{r}}_{o}^{3}=kt, \end{array}$$

where $\overset{-}{r}$ is the average particle radius, ${\overset{-}{r}}_{o}$ is the average initial radius, *k* is the rate constant, and *t* is time. The rate constant *k* is given by

(4) $$\begin{array}{c} k=\frac{8\gamma V_{m}^{2}c_{r=\infty }}{54\pi \eta aN_{A}}, \end{array}$$

where *γ* is the surface energy, *V*_*m*_ is the molar volume, *c*_*r*=*∞*_ is the equilibrium concentration at a flat surface (i.e., the bulk solubility), *η* is the viscosity of the solvent (at room temperature, *η*_water_ = 8.94 × 10^−4^ Pa·s, *η*_absolut  ethanol_ = 10.74 × 10^−4^ Pa·s and *η*_iso  propanol_ = 19.45 × 10^−4^ Pa·s [19]), and *a* is the solvated ion radius.

From (4) it is apparent that the rate constant *k* is inversely proportional to *η* if *c*_*r*=*∞*_ and *γ* are independent of the solvent. The viscosity of water is several times higher than the viscosity of alcohols such as ethanol or isopropanol, and hence the coarsening process of nanostructures in water takes place much faster and results in big agglomerated clusters.

In addition, the polarity of water is also more favorable for the formation of large agglomerated clusters. The idea that polarity of solvent can alter the morphology of nanoparticles was reported theoretically and experimentally by the group of Leekumjorn [20]. In their report, the authors showed that solvent polarity has a better correlation with the simulation and experimental results than other parameters such as dielectric constant or dipole moment. Computational results showed that the nonpolar solvents of hexane, toluene, and benzene (polarity index *E*_*N*_^*T*^ < 0.120) kept oleate-capped nanoparticles in suspension and solvated the oleate chains so that the oleate layer swelled to full extension. In contrast, as the most polar solvent tested (*E*_*N*_^*T*^ = 1.000), water caused nanoparticles to aggregate and precipitate. For solvents of intermediate polarity like ethanol, acetone, and chloroform, quantum dots were colloidally stable in solvents below a critical polarity index value (*E*_*N*_^*T*^ = 0.307).

Some other organic solvents rather than water have been also tested for the synthesis of CuO nanostructures. Different alcohols will then be the next choice to synthesize copper oxide nanoparticles due to their excellent characteristics as solvents. Alcohols have a very polar hydroxyl (OH) group, with the high electronegativity of oxygen allowing hydrogen bonding to take place with other molecules. Also, alcohols have nonpolar carbon chains; therefore, they can dissolve both polar and nonpolar substances, while water can dissolve only polar ones. Alcohols are low toxic, and ethanol is the least toxic of the alcohols, which makes it more suitable for use in industry and consumer products. The most common used alcohols are ethanol (boiling point 78.4°C), glycerol (290.0°C), propanol (97°C), propylene glycol (188.2°C), and ethylene glycol (197.3°C). Another advantage of alcohols is that they could be removed easily during the washing process without leaving unwanted impurities on the products. Because in liquids phase process, CuO nanoparticles were formed through the decomposition of Cu(OH)_2_, organic solvents of boiling points higher than the decomposed temperature of Cu(OH)_2_ should be chosen. However, copper hydroxide is decomposed into copper oxide at a quite low temperature, which is only 80°C; then most of alcohols fulfill those criteria and can be utilized to prepare CuO nanostructures. Even ethanol, of which boiling point (78.4°C) is slightly lower than the decomposed temperature of Cu(OH)_2_, could be used. However, following the above discussion, as the carbon chain gets longer, alcohols become less polar and have higher viscosity; it is more favored for synthesis of small, uniform, and nonaggregated nanoparticles.

Some other organic solvents such as, dimethylformamide (DMF) and dimethyl sulfoxide (DMSO) were also used but only in a few publications and the influence of solvent on the size and shape of the product was not well discussed.

#### 2.1.2. Salt and Alkali Metal Solution

In principle, any kind of soluble copper salts could be used as precursor to prepare CuO nanostructures without much difference or at least there seems to be no report on the influence of copper salt precursor. Various copper salts such as chloride, nitrate, sulfate, acetate were used to prepared CuO nanomaterials, however the effect of different copper salts were not discussed in details. However, particle size and uniformity of copper nanoparticles prepared from copper acetate seem better than those from inorganic copper salt. A reasonable explanation is that carboxylate groups are still adsorbed on the surface of the copper oxide nanoparticles and play the role of a surfactant and suppress nanoparticles from growth and aggregating process.

The other main necessary precursor for synthesizing CuO nanoparticles is the base agent which provides hydroxyl ion to react with copper salt and gives the Cu(OH)_2_ precipitation. The most common used precursors are sodium hydroxide and potassium hydroxide. NaOH seems to be more preferred just because it is much less expensive than KOH, while both compounds give almost the same effect due to their similar properties. NH_4_OH could also be utilized; however, the high volatility of NH_4_OH brings some limitation during the synthesis process and, hence, NH_4_OH appears only in few reports of preparing CuO nanoparticles. Sun et al. reported that using ammonia may enhance the agglomeration of the products due to the high polar nature of ammonia [21].

Mole ratio between copper ion and OH^−^ group should be 1 : 2 according to (1), (2); however, many salts of Cu readily hydrolyze in water and thus induce high solution acidity (pH < 2), while pH can play an important role in the dynamic process during the reaction. Hence, the Cu^2+^/OH^−^ ratio in the precursor solution could be adjusted from report to report in order to obtain the nanoproduct of desired morphology and size.

A slower reaction rate, which leads to small size and narrow size distribution of the products, can be achieved by using low concentration of reactants. However, if the concentration of Cu salts was too low, the amount of the CuO product would become negligible. Concentrations that were too high, on the other hand, make the product agglomerate so the concentration of precursor solution should be chosen carefully to balance between the quantity and quality which refers to small size and good separation of the nanoproduct. Limitation of using low concentration solution could be an obstacle to the mass production of nanoproduct due to the wastage of solvent, but recycle of the used solvent could be a solution for such problem.

#### 2.1.3. Surfactant

After the nucleation and growth process are completed, the average size of CuO nanoparticles could continue to increase due to agglomeration. This process reduces the quality of CuO nanoparticles in particular and nanoproducts in general, so restraining the particles from self-aggregating is a very important task.

As the nanoparticles were agglomerated, it is challenging to separate them. In nanostructure fabrication and processing, it is difficult to prepare small nanoparticles due to the challenge in huge surface energy; then preventing the as produced nanoproducts from aggregating together is also a real problem that is needed to be solved. There are two main stabilization mechanisms: electrostatic and steric stabilization. Electrostatic stabilization keeps the system at kinetic equilibrium while steric stabilization keeps the system at thermodynamically stable case.

Electrostatic stabilization is based on the repulsion between equally charged particles. By considering the combination of van der Waals attractions and electrostatic repulsion Derjaguin, Landau, Verwey and Overbeek successfully proposed a theory to describe the electrostatic stabilization of colloidal nanoparticles. Even though some of the assumptions in the DLVO theory [22], which is named after the scientist, such as infinitely flat surface or constant charge density of the particles, were far from reality, it explained well interaction between two approaching particles and hence is widely accepted by the science comunity.

However, there is no report of using electrostatic solely to stabilize the CuO nanoparticles prepared in liquid phase. Instead, most groups used various surfactants to provide the steric barrier or to combine both mechanisms to achieve the best result in preventing the CuO nanoproduct from aggregation. Normally, surfactants have a hydrophilic head and a hydrophobic tail. The polar heads of surfactants are absorbed on to the surface of nanoparticles, while the hydrophobic tail provides the steric repulsion to stop agglomeration. To provide sufficient repulsion between nanoparticles, the length of the stabilizer needs to be significantly longer than the characteristic size of the nanoparticles and also the polar head must have a tight bonding with nanoparticles. That is the reason why stabilizers are often high molecular weight polymers. Depending on the nature of each different polymer, another beneficial effect that surfactant may bring is to increase viscosity of the liquid media and thus minimize the rate of coarsening, as presented in the previous part.

It is extremely useful if both stabilization mechanisms above were achieved at the same time. Many groups had taken advantages of such combination by using certain polymers, of high ion density. The ionic nature of the polymer results in the electrostatic repulsion between nanoparticles while the long polymer chain simultaneously keeps the nanoparticles away from each other by steric effect. Among the polymers of this kind, polyvinylpyrrolidone (PVP) is one of the most commonly used as it can be dissolved in both polar and nonpolar solvents; the polar amide group in polymeric chain of PVP is readily attached to the surface of nanoparticles to protect them from aggregation by the two stabilization mechanisms. When dissolved into a solvent, these polymers will form a network, which can also play as templates to guide the growth of nanoproducts. It is then the network formed by polymer that could alter the morphology of the nanostructures.

Although polymer stabilizers are introduced primarily to form a layer on the surface of nanoparticles, occupy the growth sites, and reduce the growth rate of nanoparticles so as to prevent agglomeration of nanoparticles, the presence of such polymer stabilizers during the formation of nanoparticles can have various influences on the growth process of nanoparticles. Interaction between the surface of a solid particle and polymer stabilizer or stabilizer with each other may vary significantly depending on the surface chemistry of solid, the polymer, solvent, and temperature. The complicated dynamic process of the interaction between polymer and nanoparticles leads to a strong dependence of morphology and size of nanoparticles on type of surfactants or even the amount of surfactant that was used. Further increasing the amount of polymer in the reaction mixture was found usually to increase the sphericity of the particles. It can be easily understood by considering the fact that increased amount of polymer produces steric resistance for the diffusion and consequently results in a diffusion controlled growth, which favors the formation of spherical particles.

### 2.2. Solution Methods to Prepare CuO Nanostructures

CuO nanoproducts could be prepared by various methods such as sol-gel, spray pyrolysis, precipitation, solvothermal or sonochemical methods. Each method more or less has some limitations; for example, solvothermal could be used to prepare material in extreme conditions such as high temperature, high pressure; the bad side is that it takes quite long time, sol-gel process is quite complicated, and there are so many parameters need to be controlled. Among the methods that could be applied to synthesize nanomaterials, microwave irradiation method which is a chemistry physics combined method currently shows its many advantages and cut down the number of limitations, which are usually brought by other methods.

Microwave energy is a very efficient means of heating. Chemical reactions that took long time to complete can now be accomplished in minutes with the aid of microwave. Microwave assisted synthesis not only helps in implementing green chemistry but also led to the revolution in organic synthesis. Microwave irradiation is well known to promote the synthesis of a variety of compounds, where chemical reactions are accelerated because of selective absorption of microwave by polar molecules. It was found that the main advantages of microwave irradiation method compared with conventional method are fast, mild, energy-efficient, and friendly with the environment. The effectiveness of microwave irradiation in the preparation of nanoparticles can be explained by the preeminent advantages of microwave as a means of heating. The mechanism of traditional heating process is conductive heat or heating by convection currents and hence this is a slow and energy inefficient process due to the energy lost at the wall of the vessel. Normally the wall of the vessel absorbs heat first; then heat is transferred to the liquid inside so the temperature of the outside surface needs to be in excess of the boiling point of liquid for the temperature inside the liquid volume to reach boiling point. These disadvantages can be overcome easily by applying microwave technique because when heating with microwave, vessel wall is transparent to microwave and solvent/reagent absorbs microwave energy directly. The direct in core heating and instant on-off pulse of heat lead to the formation of a homogenous temperature gradient and reduce the time reaction. These advances in turn produce smaller particles of uniform size and shape. By using microwave irradiation, different nanostructures of CuO of uniform size and shape could be prepared in a few minutes [16–18].

## 3. Fundamental Properties

### 3.1. Crystal Structure and Some Physics Constants

**Figure 2:**
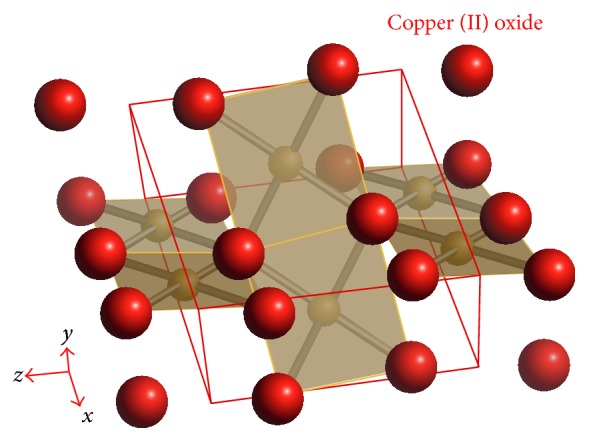
Crystal structure of CuO.

CuO crystal has monoclinic structure (Figure 2) and belongs to C^6^_2h_ symmetry. Cupric oxide has four formula units per unit cell. The coordination number of copper atom is 4, which means that it is linked to four oxygen neighbor atoms in an approximately square planar configuration in the (110) plane. In all crystallized solids, divalent copper surroundings are always very distorted by a strong Jahn-Teller effect which often leads to more stable square planar groups.

The Cu-O bond lengths in this plane are 1.88 and 1.96 Å, respectively, which are larger than those in the cuprous oxide [23]. The next two Cu-O bond lengths perpendicular to the plane are much greater, so an octahedral type of coordination can be ruled out. The O atom is coordinated to four Cu atoms in the form of a distorted tetrahedron. It is often accepted that the CuO has a mixture bonding of ionic and covalent bonding, even though the oxidation state of Cu in CuO is unquestionably Cu^2+^.

The lattice parameters of cupric oxide [24] are *a* = 4.6837 Å, *b* = 3.4226 Å, *c* = 5.1288 Å, *β* = 99.54°, and *α* = *γ* = 90°. Some other basic physics constants of CuO were also summarized in Table 2.

X-ray diffraction is obviously the most common tool to study the crystal structure of materials and confirm the purity of the product. From X-ray diffraction pattern, the lattice constants, lattice strain, or particles size can be extracted following the Debye Sherrer formula. According to Aparna et al. [12], elastic strain calculated from XRD results shows that CuO nanoparticles smaller than 20 nm have high strain and greater particles have less strain. This phenomenon relates to the pressure different between the inside and outside of a curved surface, resulting from the surface tension.

**Figure 3:**
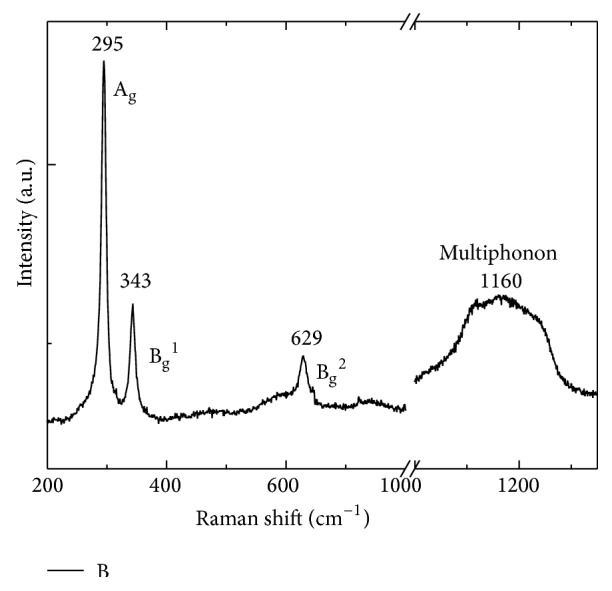
Raman spectra of CuO nanostructures prepared by microwave irradiation method (author's data).

Raman spectroscopy, which is a sensitive probe to the local atomic arrangements and vibrations of the materials, has been also widely used to investigate the microstructural nature of the nanosized materials in general and CuO nanomaterial in particular. Raman scattering also provides useful information about the structures and bonds of materials. Raman scattering could help to detect the existence of unintended phases such as Cu_2_O or Cu(OH)_2_ or show the crystallinity of the product. The space group of CuO is C^6^_2h_ with two molecules per primitive cell so the zone center Raman active normal modes of CuO are Γ_RA_ = 4A_u_ + 5B_u_ + A_g_ + 2B_g_. Among these vibration modes, there are three acoustic modes (A_u_ + 2B_u_), six infrared active modes (3A_u_ + 3B_g_), and three Raman active modes (A_g_ + 2B_g_). Three well known bands of CuO are A_g_ (296 cm^−1^), B_g_^(1)^ (346 cm^−1^), and B_g_^(2)^ (631 cm^−1^) [13].  Figure 3 shows Raman spectra of CuO nanostructures prepared by microwave irradiation method with three typical modes.

Xu et al. studied Raman spectra of CuO nanocrystals with different grain sizes at room temperature and high temperatures up to 873 K [25]. They reported that Raman intensity is related to the grain size. Samples of smaller grain size show stronger and sharper Raman peaks which also shift to smaller wavenumbers. The red shifts could be explained by the phonon confinement effect in nanometer size materials [25, 26].

It should be noted that crystal defects, of which number increases rapidly as the grain size decreases due to the large surface/volume ratio, could contribute significantly to Raman spectra as all of the three Raman modes in CuO relate only to the vibration of oxygen atoms as was pointed out by Irwin and Wei [27].

Apart from the three main vibration modes above, Wang et al. [28] reported multiphonon band of CuO nanostructures, which appears at wavenumber of 1130 cm^−1^ and relates to the inharmonic coupling between phonons in polar solid. In particular, the multiphonon band 2B_g_ in CuO was suggested to be the stretching vibration in the *x* ^2^-*y* ^2^ plane, induced by the electronic density variation in this layer. The intensity of multiphonon Raman peak is much weaker than that of the one phonon band and varies with morphology and the size of the as prepared CuO nanostructures. The authors reported that the multiphonon band of the as prepared CuO nanostructures with belt-like morphology possesses higher intensity than that of the CuO nanostructures with shuttle-like morphology, while Raman intensity of multiphonon band of the shuttle-like morphology is higher than that of the CuO nanostructures with bamboo leaf-like morphology. The difference in the Raman intensity of different morphology was explained by anisotropy of different nanostructures. The electronic movement along the *x*-*y* plane becomes significant in *x*-*y* plane and promotes the intensity of 2B_g_ mode in belt-, shuttle-, and bamboo leaf-like nanostructures. Another explanation for the variation in the Raman intensity of this mode is the phonon-plasmon coupling due to high local density of anisotropic carriers in CuO nanostructures. The variation in the multiphonon intensity shows a finite size and crystallinity effect of CuO nanostructures.

### 3.2. Optical Properties

Compared with other properties such as electrical conductivity and field emission, optical property of CuO nanostructures has been much less investigated and discussed so far. As a p type semiconductor, a narrow bandgap of around 1.2 eV was reported for bulk CuO [3]. In fact, reported values of bandgap for CuO were not in good agreement; for example, bandgap in the range of 1.56 and 1.85 eV was reported for CuO thin films [29, 30]. In addition, the variation of bandgap could also relate to quantum size effect in different CuO nanostructures [31, 32].

For nanomaterials, several methods could be applied to characterize the optical properties or to estimate the bandgap in particular. Among these methods, UV-vis absorption spectroscopy, as a nondestructing and quick technique, is one of the most convenient methods to reveal the energy structures and optical properties of semiconducting materials. The optical bandgap of semiconductor material can be calculated from the absorption spectra by using Tauc's relation [33]

(5) $$\begin{array}{c} \alpha h\upsilon ={\left(h\upsilon -E_{g}\right)}^{n}, \end{array}$$

where *hυ* is the energy of incident photon and *n* is the exponent factor that determines the type of electronic transition causing the absorption and can take the values 1/2 and 2 depending whether transition is direct or indirect, respectively. Bandgap is determined from the intercept of the straight line with the horizontal axis. By UV-vis studies, some groups have reported significant blue shift (up to 1.7 eV) [34] in the absorption edge compared with the bandgap of bulk material, which was explained by the quantum confinement effect in these nanostructures.

In literature, optical behavior of CuO nanomaterials has been mainly assessed by absorption techniques, while luminescence techniques, which are important tools to investigate electronic transitions in semiconductors—including band edge or near band edge transitions—have been seldom used. The low emission efficiency of CuO is the main reason for the lack of luminescence data for CuO nanomaterials; also the results on the origin of luminescence of CuO remain contradictory.

There are several photoluminescence bands generally reported for CuO nanostructures which expand from UV to near IR region; however the most frequent peaks fall in the region from 400 to 600 nm. Generally, the deep level emission in CuO consists of a green emission at around 605 nm and a near-yellow emission at around 680 nm. Though the origin of deep level emission in CuO is under debate, and little information is available on the CuO defect structure, the deep emissions are generally supposed to relate to defects in CuO nanomaterial. CuO is intrinsically a p type semiconductor due to the existence of Cu vacancies. However, recent theoretical calculations indicate that although Cu vacancies are the most stable defects in CuO, they do not make any changes in the electronic structures of CuO. Otherwise, oxygen vacancies or O_Cu_ antisite defects are likely responsible for these emissions while their formation energy is not much different from formation energy of Cu vacancies [35]. The green emission is commonly assigned to the singly ionized oxygen vacancies; the yellow or red emission has been supposed to relate to the interstitial metal ion in the oxide. Therefore, the evolution of green and yellow bands in CuO is competitive with each other. The blue shift behavior of the near band edge transition in comparison with that of the bulk CuO in combination with the findings from UV-vis analysis was normally attributed to the enhancement of the quantum confinement effect resulting from the decrease in the dimensional structure and the size of the nanoparticles. One can also evaluate the concentration of structural defects by comparing the photoluminescence intensity ratio of near band edge emission to green deep level emission. This kind of assignment was applied by some groups. Vila et al. [36] observed luminescence bands centered at 1.33, 1.23, and 1.11 eV in CuO nanomaterial and suggested that the emission with highest energy corresponds to near band edge transition in CuO while the two other emissions are most probably introduced by oxygen vacancies and oxygen on copper antisite defects [35, 37, 38].

Besides emissions in visible or IR region, other authors announced emissions in the UV region. Santra et al. [29] observed the near band edge emission at 395 nm in the violet region. Aslani [39] reported near band edge emission at 300 nm. Mageshwari and Sathvamoorthy [40] reported several photoluminescence peaks at 325, 339, and 356 nm and explained the difference photoluminescence emission peaks of CuO as agreed with earlier reports by various sizes and shapes of CuO nanostructures. This fact indicates that luminescence properties of CuO are strongly dependent on the morphology of the nanocrystals.

Gizhevskiǐ et al. [41] studied the influence of temperatures and annealing time during sample preparation on the photoluminescence properties of CuO nanocrystals and reported three main broad emission bands centered at about 305 nm (4.07 eV), 505 nm (2.46 eV), and 606 nm (2.05 eV) [4, 14, 39, 42, 43]. The 2nd band was attributed to band edge emission while the band at 600 nm region was also assigned to defect related states. The emission at the band edge contains several subpeaks due to the band edge emission from Γ_1_^+^ to the new sublevels which might arise by the interaction of two excitons or the ^3^D-^1^D splitting in Cu^+^ (3d^9^ 4s^2^) at 300 K. PL intensities of the 305 nm (4.07 eV) band were seen to increase with the increasing temperature treatment and were explained by the enhancement of the crystallinity of the sample.

### 3.3. Magnetic Properties

As mentioned in the section of optical properties, photoluminescence and magnetic properties are aspects of least study for CuO nanomaterials; however CuO nanostructures showed interesting and unique magnetic properties so it is worth making a summary on this topic. CuO, which is different from other antiferromagnetic transition metal monoxides such as NiO, MnO, and CoO, shows magnetic order even above its Neel temperature.

Kimura et al. [44] reported that the ferromagnetic or antiferromagnetic ordering in CuO single crystal could be controlled by fine tuning the bond angle between in plane Cu-O-Cu. Large bond angle Cu-O-Cu was believed to result in large super exchange interaction, favored for antiferromagnetism while spiral ordering in certain crystallographic directions favors a ferroelectric phase in the same material. Due to the complex dependence on temperature of spin structure, CuO normally shows two antiferromagnetic transitions at 213 K and 230 K, respectively. The first Neel temperature related to commensurate to incommensurate transition, whereas the second one is attributed to incommensurate to paramagnetic transition.

A hysteresis loop or a bifurcation in FC-ZFC curves was reported by a few groups as evidences for ferromagnetism in CuO nanostructures [45]. For CuO nanomaterials, some reports showed that magnetic properties could vary, depending on size of the nanostructures. For example, Punnoose et al. [46] showed that susceptibility of CuO nanoparticles is inverse proportional to the particle size for particle smaller than 10 nm. The magnetic behaviors of CuO nano particles larger than 10 nm are similar to bulk material. However, morphology of CuO nanostructures should play an important role as well, because other authors showed that ferromagnetism could also arise in nanosheets, nanoneedles, and so on of larger size [47, 48]. Hysteresis loop of weak ferromagnetism was observed at 5 K in CuO nanosheets prepared by hydrothermal synthesis by the group of Zhao et al. [47]. Temperature dependence of magnetization showed Neel temperature of CuO nanosheets of 219 K while the FC and ZFC data show obviously maxima at about 40 K, which probably corresponded to blocking temperatures. Below 40 K, the magnetization increases rapidly in FC data which reconfirms the existence of weak ferromagnetism in CuO nanosheets. The influence of impurities such as Fe, Ni at low content (0.5%) was excluded as the most probable ferromagnetic material is CuFe_2_O_4_ but the magnetization of this phase is only half of as-measured magnetization of CuO nanosheets. The ferromagnetism resulted from the uncompensated spins on the surface of nanomaterials, which will be orientated under magnetic field and results in weak ferromagnetism as observed in CuO nanostructures. As the surface area of nanomaterials is much larger than that of bulk CuO, the surface effect, which is in this case shown by the ferromagnetic property, becomes dominant and hence CuO nanomaterials will exhibit ferromagnetic property more clearly. This argument could explain the size and morphology dependence of the ferromagnetism of CuO nanostructures.

According to Bhalerao-Panajkar et al. [48], the core shell nature of their CuO nanoparticles with a ferromagnetic shell and antiferromagnetic core may be responsible for bifurcation of FC-ZFC curves because in pure antiferromagnetic or dominantly antiferromagnetic particles no bifurcation in FC-ZFC curves occurs.

The bifurcation in FC-ZFC could also arise due to the ferromagnetic nature of the shell supporting the core shell nature of the particles. Ferromagnetic/antiferromagnetic core shell structure was also supported by the asymmetry of the coercivity plot, which indicates the presence of an exchange field in core/shell system as suggested by other groups.

## 4. Applications of CuO Nanoparticles

CuO first attracted attention of chemists as a good catalyst in organic reactions but recently discovered applications of CuO such as high-Tc superconductors, gas sensors, solar cells, emitters, electronic cathode materials also make this material a hot topic for physicists and materials science engineers. Some of the most interesting applications of CuO nanomaterials are sensing, photocatalyst, and super capacitor that will be highlighted in this section.

### 4.1. Sensing Applications

It is surface conductivity that makes CuO an ideal material for semiconductor resistive gas sensor applications and in fact CuO nanomaterials were used for detection of many different compounds such as CO, hydrogen cyanide, and glucose. As sensing properties closely relate to the chemical reaction on the surface of sensor, the specific area is a key factor to achieve high sensitivity sensor. Due to the high surface area/volume ratio, the sensing property of CuO nanomaterials was enhanced greatly. The shape of CuO nanostructures was also believed to affect significantly the sensing properties of CuO nanomaterial; for example, spherical crystals often show higher sensitivity than columnar one.

Aslani and Oroojpour[4] studied CO-sensing properties of different CuO nanoparticles prepared by solvothermal route as a function of morphology and size of nanoparticles. The results show that cloud like structures with high surface area/volume have higher response and detection limit than other morphologies. Yang et al. [5] also showed that the specific surface area of these CuO nanostructures plays an important role in the sensitivity for detecting HCN. Both sides (5 mm in diameter) of a silver-coated quartz crystal microbalance (QCM) resonator were covered with CuO nanostructures; the resonator was used as sensing probe in a quartz crystal resonator. The absorbance of HCN gas on sensor is indicated by the shift of resonant frequency. As specific area of CuO nanostructure used for coating the probe changes from 9.3 m^2^/g to 1.5 m^2^/g, the sensitivity reduces from 2.26 to 0.31 Hz/*μ*g. In both reports, the authors showed that the sensitivity of sensors depends not only on the surface area but also on the morphology of the nanostructure. The change in sensitivity of different nanostructures could be explained by the variation in the chemical reactivity of different crystal planes.

Glucose detection is another important application of CuO in sensing field. In conventional methods, glucose detection is based on the use of glucose oxidase which is an enzyme used in the sensor. This enzyme catalyzes the oxidation of glucose to gluconolactone and simultaneously produces H_2_O_2_. Glucose level is then evaluated by estimation from electrochemical response to the liberated H_2_O_2_. However, the main disadvantages of conventional methods are high cost and lack of enzyme stability, complicated immobilization procedures of enzyme, and the coexisting interferences in the biological fluids together with critical operating conditions. Most of those limitations could be solved by using CuO nanostructures as an alternative oxidase, where CuO nanomaterials act as catalyst to convert glucose into gluconolactone and finally to glucose acid. The better efficiency of the oxidized reaction in CuO based sensor resulted from high surface area, surface energy which enhanced electron transfer ability of CuO nanomaterials.

### 4.2. Supercapacitors and Electrodes for Lithium Ion Batteries

Pseudocapacitors also known as one type of supercapacitors have attracted significant attention of researcher as efficient energy storage devices with superior properties such as high power density, excellent reversibility, and long cycle life time-dependent power, which are necessary properties of electronics portable devices. As the demand for high capacity energy storage in modern life was raised continuously, pseudocapacitors have become a hot topic recently. Among transition metal oxides which are considered as ideal electrode materials for pseudocapacitors, CuO is a really promising candidate for its abundant resources, environmental compatibility, cost effectiveness, and favorable pseudocapacitive characteristics.

It was found that the morphology and particle size of CuO remarkably affected its specific capacity. Cauliflower like, nanobelt-shaped, and feather-like CuO nanocrystals were synthesized by the chemical deposition method by group of H. Zhang and M. Zhang [49]. According to the authors, morphologies of the CuO nanostructures can influence the electrochemical properties significantly. The electrochemical properties of CuO as electrode material were enhanced by the improving of morphology. Cauliflower-like CuO exhibited a higher specific capacitance (116.9 F g^−1^) than nanobelt-shaped and feather-like CuO and also showed good reversibility. Specific capacitance of cauliflower-like CuO (115.3 F g^−1^) was 343.5% higher than CuO bought (26 F g^−1^) at 5 mA cm^−2^. The CuO cauliflower-like exhibited a higher utilization efficiency and better property for electrolyte diffusion than the feather-like and nanobelts structures. The increasing order of the specific capacitance was consistent with increasing sequence of CuO specific surface area, indicating that the highly mesoporous structure and high specific surface area of the electrode facilitate the ions to transfer into the porous structure more easily which would lead to more redox faradic reactions and surface adsorption of electrolyte cations.

CuO nanomaterials could substitute for graphite anode in LIBs due to its superiorities such as high theoretical capacity (670 mAhg^−1^), improved safety, low cost and environmental benignity. However, it also suffers very rapid capacity decay caused by huge and uneven volume variations (around 174%) during the lithium uptake/releasing process. One possible approach to improve the electrochemical performance of CuO materials is to use well-configured nanostructures ranging from zero-dimensional nanoparticles to multidimensional assemblies. In these nanostructures, not only lithium diffuses much easier, but also the strain associated with lithium uptake could be well accommodated, leading to better electrochemical performance. Wang et al. [50] successfully prepared nanorods and nanosheets on a Cu substrate. The unique nanostructural features endower them excellent electrochemical performance with high capacities of 450–650 mAh g^−1^ at 0.5–2 C and almost 100% capacity retention over 100 cycles after the second cycle. Recently composite material of CuO nanomaterial was developed to further increase the capacities of LIBs. Rai et al. [51] successfully used CuO/reduced graphene oxide nanocomposite as anode materials for lithium ion batteries. The initial discharge capacity of the pure CuO nanoparticles and their nanocomposite is 785.2 mAh g^−1^ and 1043.3 mAh g^−1^ with reversible capacity retention of 392.1 mAh g^−1^ and 516.4 mAh g^−1^ after 45 cycles, respectively.

### 4.3. Photocatalyst and Solar Energy Conversion

Water pollution due to organic wastage from industry production has become a serious problem in the world today. Most of organic compounds in waste water are toxic and cannot be decomposed naturally so they need to be treated with care before disposal. Water treatment using semiconductor catalysts under solar UV or visible light seems to be the most effective way as it has shown that this method could be employed to totally decompose many different organic compounds into biodegradable without complex technologies. CuO is a promising candidate due to low cost and abundance.

As a p type semiconductor of narrow bandgap in visible region, CuO is expected to be a good material for application in photocatalyst and solar energy conversion. However, some groups reported that CuO shows almost no or very little photocatalyst properties under visible light. Adding some amount of H_2_O_2_ could help to greatly improve the photocatalyst efficiency under visible light. Yecheskel et al. study the degradation of brominated flame retardants by copper oxide nanoparticles and saw that adding an amount of H_2_O_2_ enhances the photocatalyst properties of CuO nanoparticles [1]. They also showed that the interaction of CuO nanoparticles with H_2_O_2_ results in an electron spin resonance spectrum similar to spectrum of Cu^2+^ ion. This fact might indicate a release of Cu^2+^ ion to the solution or changes in the electron configuration of CuO nanoparticles in the solid phase. Based on these effects, the authors suggested that H_2_O_2_ may have a role in the activation of CuO catalyst besides being an oxidative agent. It is noteworthy that photocatalyst properties also show dependence on size and shape of CuO nanostructures which again can be explained by the enhancement due to large surface area as well as the anisotropic of single crystals nanostructures of CuO, meaning that the photocatalyst of different crystal plane in CuO could be different.

CuO could also be good candidate in solar energy conversion due to many properties: high absorption coefficient, narrow bandgap in visible region which is expected to give high conversion efficiency, being nontoxic, stability, good electrical conductance, simple manufacture process, and so on. A more direct way to convert solar energy to electricity is to use CuO as absorber in solar cell. Efficiency of solar cell based on CuO is far lower than efficiency of leading chalcogenide system such as CIS or CIGS, but due to its low cost, abundant resource, and simple preparation process it was shown that efficiency of only several percents in cell based on CuO is good enough to make commercial solar cells. Different from its counterpart Cu_2_O, CuO is used less for solar cell as the achieved efficiency for Cu_2_O is higher. Number of reports on CuO solar cell is rare but recent results show very promising achievement, which shows that further development of CuO nanomaterials based solar cell has a bright future. Kidowaki et al. [52] prepared solar cells based on CuO nanoparticles/C60 junction which provided efficiency *η* of 1.8 × 10^−6^%, fill factor of 0.25, *J*_sc_ of 0.18 × 10^−3^ mA cm^−2^ and *V*_oc_ of 0.04 V. A crystallite size of CuO was determined to be 3.4 nm, and higher crystallinity of CuO would increase the efficiency of the CuO/C60 solar cells. Or more recently, using solvothermal method, Chandrasekaran [53] prepared CuO nanoparticles and used the product to make a solar cell with efficiency of 0.863%, which is compared with other reported values [15, 54, 55]. Up to now, the record efficiency of solar cell based on copper oxide is about 2%, while the theoretical value is about 20%, so efficiency of several percents is obviously achievable.

CuO nanomaterials could also be used as good substitution for expensive noble metal cathode in dye solar cell. This topic was first introduced by Anandan et al. [56] in 2005 and the optimal power conversion efficiency when using CuO nanorods as electrode was 0.29% compared with 1.23% when using Pt as electrode in the same condition. By using CuO nanoneedles of higher surface active area, Liu et al. [57] obtained an efficiency of 1.12%, for TiO_2_ based dye solar cell. This result shows that nanomaterials of CuO could replace well Pt electrodes and can even give better efficiency under optimization process.

### 4.4. Field Emission Effect

Field emission displays are now in a more dominant position in the market compared with CRT displays because of their advanced properties such as high brightness, good color rendition, short response time, and low power consumption. Among the various nanomaterials studied for field emission applications, 1D nanostructures of CuO emerged as very promising field emitters because of some advantages: low turn-on field, high current density, and low fabrication cost [58–62].

Liu et al. [58] investigated the field emission properties of an individual CuO nanoneedle by in situ microscopy. The authors showed that individual nanoneedle possesses good field emission properties, such as low turn-on field of 5.3 V/*μ*m, high maximum current of 1.08 *μ*A at 9.7 V/*μ*m. The field emission properties of the single CuO nanoneedle and CuO nanoneedle's film arrays are also compared and the results showed that the screening effect played a key role in the field emission properties.

Hu et al. [60] used a simple method of direct heating of bulk copper plates in air to obtain CuO nanowire films on a large scale. The length and density of nanowires could be controlled by growth temperature and growth time. The as produced CuO nanowires have high density, good preferred orientation, and sharp tip, which is very beneficial to field emission. Field emission measurements showed that CuO nanowires have a low turn-on field of 3.5–4.5 V/*μ*m^−1^ and a large current density of 0.45 mA cm^−2^ under an applied field of about 7 V*μ*m^−1^. The authors also showed that CuO nanowires having large length/radius ratio can effectively improve the local field, which enhance field emission. Zhu et al. [61] prepared some different CuO nanostructures by solution method. By varying the oxidant concentration, the authors can modulate the morphology of the nanoproducts from nanorod to nanotubes. The tip morphologies of CuO nanostructures were found to be crucial for the field electron emission, and the nanorods with needle-like tips showed superior emission properties with a turn-on field of 3.5 V/*μ*m and a field enhancement factor of 2107, compared to other structures.

Apart from improving the field emission efficiency by optimizing the aspect ratio (length/diameter) of 1D nanostructures, some other methods were also utilized to enhance the field emission current. Wang and Li [62] found that laser irradiation could effectively enhance the field emission current of CuO nanowire arrays. The effects of laser intensity, wavelength, emission current, and working vacuum on the enhancement have been investigated in detail. Among these factors, the contribution from extra excited electrons, which increases the number of electrons in conduction band of CuO for subsequent tunneling, is dominant. The observed laser induced enhancement in field emission current is attributed to the interplay of two factors, namely, laser induced electron transition to excited states and surface oxygen desorption. Based on the idea of light induced field emission of their work, new vacuum nanodevices of CuO nanowires such as photodetectors or switches could be developed in the future. Another example is the work of Maji et al.[34], where the authors also prepared CuO nanowire arrays by thermal oxidation. In order to improve the field emission properties of CuO nanowires they coated a ZnO layer on a Cu substrate before the thermal oxidation process. The ZnO layer was deposited by immersing a Cu foil into an aqueous solution of zinc nitrate and hexamethylenetetramine at 95°C for several hours. The turn-on field of the ZnO-coated CuO nanowire array was 0.85 V/*μ*m compared with turn-on field 6.5 V/*μ*m of CuO nanowires without ZnO coating layer at the same current density of 10 *μ*A/cm^2^. The authors suggested that in addition to the enlarged nanowire density and aspect ratio, crack elimination may be the reason for the enhancement of field emission properties.

## 5. Conclusion

In conclusion, CuO nanostructures have been widely studied and are receiving more and more attention from material scientists and engineers recently because of their interesting properties and potential applications in various fields. In this study, we make a summary on the influences of different factors of synthesis process, some unique properties, and some promising applications of CuO nanostructures. We focus on the some chemical synthetic strategies along with associated influence of basic factors of synthesizing process for CuO nanostructures, as well as their interesting fundamental properties, and interesting applications. Understanding the synthesizing process as well as the characteristics of CuO nanostructures is fundamental for further purposes to realize application of CuO nanostructures in daily life and technology. Some unique properties of CuO nanostructures which make CuO different from other transition metal oxides were also summarized and highlighted. Some potential applications based on CuO nanostructures are also presented.

Although encouraging developments and fascinating achievements in CuO nanostructures have been obtained as overviewed in this paper, better understanding for controlling morphology, structures, and properties of cupric oxide nanostructures and finding ways to take advantages of these interesting properties of such nanostructures still require much effort from scientists but also bring in opportunities for further development.

## Figures and Tables

**Table 1:** Summary on the effect of starting materials, solvents, and surfactants on morphology of CuO nanostructures.

Morphology	Size	Solvent	Starting materials	Surfactant	Method	Reference
Hierarchical superstructure	Diameter: 200 nm; length: 600 nm SEM	Distilled water	Cu(CH_3_COO)_2_ NaOH	Ethylene diamine-te tra-acetic acid disodium	Sonochemical	[11]

Cubic	230 nm	Water	Cu(CH_3_COO)_2_ NaOH	Without surfactant	Microwave	[18]
Sphere	40 nm, 90 nm, and 140 nm (as the concentration of salt increases)	Ethylene glycol		PVP, CTAB		

Nanoparticle	50 nm XRD	Water	CuCl_2_, NH_4_OH	Thiourea	Chemical and annealing	[63]

Nanoparticle	5–8 nm TEM and XRD	Ethanol	Cu(CH_3_COO)_2_ NaOH, methanol, and NH_4_OH	No	Alcothermal	[64]

Nanoparticle	44 nm (XRD) 80 nm (TEM)	Deionized (DI) water	Cu(NO_3_)_2_	Citric Acid; ethylene glycol	Sol-gel	[65]

Nanorod	Diameter: 50–100 nm Length: microns	2-Propanol	Cu(NO_3_)_2_	No	Solvothermal	[50]
Nanosheet	Width: 1-2 mm; thickness: 20 nm	Ethanol				

Nanoparticle	22 nm	Deionized water	CuSO_4_	Ascorbic acid NaBH_4_	Chemical reduction	[66]
	10 nm	Ethylene glycol		PVP		

Flower-like	400~600 nm	Deionized Water	Cu(CH_3_COO)_2_	No	Hydrolyzing method	[67]

Nanoparticle	3–9 nm (XRD) 11 nm (TEM)	Alcohol	Cu(CH_3_COO)_2_	No	Alcohol thermal	[68]

Nanoplatelet	Lengths: 4-5 *μ*m; thickness: 65–80 nm (SEM)	1-Butyl-3-methyl imidazolium tetrafluoroborate [BMIM]BF	Cu(NO_3_)_2_ NaOH	([BMIM]BF_4_). It serves both as solvent and as surfactant	Hydrothermal	[69]

Nanoneedle	Length ~100 nm Diameter 10–20 nm (TEM)	Water	Cu(NO_3_)_2_ NaOH	Oleic acid Sodium oleate	Coprecipitation	[70]

Nanobelt	Length: 2.5–5 *μ*m Width: 150–200 nm	Distilled water	CuSO_4_ NaOH	H_2_O_2_	Hydrothermal	[71]

Nanoparticle	100 nm (SEM)	Ethylene glycol	Cu(CH_3_COO)_2_ urea	No	Microwave	[72]

**Table 2:** Crystallographic properties of CuO and some physics constants of CuO [24].

Space group	C2/c (No. 15)
Unit cell	*a* = 4.6837 Å
	*b* = 3.4226 Å
	*c* = 5.1288 Å
	*β* = 99.54°
	*α* = *γ* = 90°
Cell volume	81.08 Å^3^
Cell content	4 CuO
Formula weight	79.57
Density	6.515 g cm^−3^
Distances	
Cu–O	1.96 Å
O–O	2.62 Å
Cu–Cu	2.90 Å
Melting point	1201°C
